# Contraception can Lead to Trophic Asynchrony between Birth Pulse and Resources

**DOI:** 10.1371/journal.pone.0054972

**Published:** 2013-01-28

**Authors:** Jason I. Ransom, N. Thompson Hobbs, Jason Bruemmer

**Affiliations:** 1 United States Geological Survey, Fort Collins Science Center, Fort Collins, Colorado, United States of America; 2 Natural Resource Ecology Laboratory, Colorado State University, Fort Collins, Colorado, United States of America; 3 Department of Animal Sciences, Colorado State University, Fort Collins, Colorado, United States of America; CNRS, Université de Bourgogne, France

## Abstract

Abiotic inputs such as photoperiod and temperature can regulate reproductive cyclicity in many species. When humans perturb this process by intervening in reproductive cycles, the ecological consequences may be profound. Trophic mismatches between birth pulse and resources in wildlife species may cascade toward decreased survival and threaten the viability of small populations. We followed feral horses (*Equus caballus*) in three populations for a longitudinal study of the transient immunocontraceptive porcine zona pellucida (PZP), and found that repeated vaccinations extended the duration of infertility far beyond the targeted period. After the targeted years of infertility, the probability of parturition from post-treated females was 25.6% compared to 64.1% for untreated females, when the data were constrained only to females that had demonstrated fertility prior to the study. Estimated time to parturition increased 411.3 days per year of consecutive historical treatment. Births from untreated females in these temperate latitude populations were observed to peak in the middle of May, indicating peak conception occurred around the previous summer solstice. When the post-treated females did conceive and give birth, parturition was an estimated 31.5 days later than births from untreated females, resulting in asynchrony with peak forage availability. The latest neonate born to a post-treated female arrived 7.5 months after the peak in births from untreated females, indicating conception occurred within 24–31 days of the winter solstice. These results demonstrate surprising physiological plasticity for temperate latitude horses, and indicate that while photoperiod and temperature are powerful inputs driving the biological rhythms of conception and birth in horses, these inputs may not limit their ability to conceive under perturbed conditions. The protracted infertility observed in PZP-treated horses may be of benefit for managing overabundant wildlife, but also suggests caution for use in small refugia or rare species.

## Introduction

**Figure 1:**
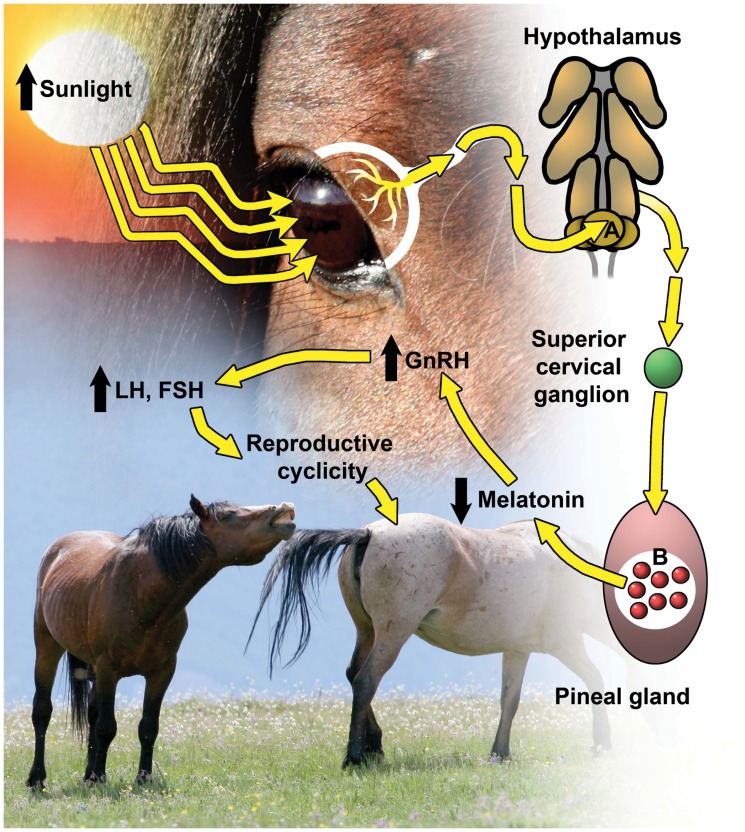
The physiological pathway from photoperiod to reproductive cyclicity in the horse (*Equus caballus*). Increasing sunlight toward the long days of summer strikes the retina, which transmits a neural signal to the suprachiasmatic nuclei (A), across the hypothalamus, and ultimately synapses to the pineal gland to stimulate release of inhibitory neurotranmitters toward the pinealocytes (B). Melatonin production then diminishes, thus increasing release of gonadotropin releasing hormone (GnRH), which increases luteinizing hormone (LH) and follicle stimulating hormone (FSH), and ultimately induces reproductive cyclicity.

Phenology of nearly all biological phenomena is influenced by natural abiotic events and is reflected in traits evolving to maximize fitness [1]. Births of many large mammal species, for example, occur in annual pulses that are regulated by seasonal cues such as photoperiod and temperature [2]. Increasing sunlight and temperature that accompanies the transition from winter to spring can trigger a physiological response in the pineal gland that initiates reproductive receptivity (Figure 1) [2]–[4]. This ultimately influences when females may conceive and thus when offspring are born. For example, feral horses (*Equus caballus*) in the northern hemisphere typically begin reproductive cyclicity in early spring and continue until late autumn; consequently, we may posit that conception should naturally peak near the longest day of sunlight (summer solstice) and parturition should peak 335–342 days later (duration of equine gestation [5]). This pattern should result in synchrony of the birth pulse with spring, when climate and forage availability for the dam can contribute to increased neonate survival.

Human actions have the potential to perturb such phenology. Resource managers are increasingly considering transient contraceptives to help limit growth of wildlife populations when species become locally overabundant and risk depleting resources or altering natural communities [6], [7]. These agents are especially appealing to managers of protected species and small populations because fertility of treated individuals may return when the transient contraception wears off [8]. However, the transient nature of many fertility control agents and variation of individual responses to those agents introduces uncertainty toward persistence of infertility and the timing of births thereafter. Cascading trophic asynchrony between birth pulses and seasonal resource peaks can have profound effects on the population ecology of wildlife species [9]–[12]. Observed changes in such phenology and their implications have garnered much attention in climate change science [13], [14], but more direct anthropogenic influences, such as fertility control, remain almost completely unstudied. Unintended persistence of contraception, and consequent phenological changes in births, may compromise the ability of populations to recover after catastrophic stochastic events and threaten viability of small refugia populations.

The immunocontraceptive porcine zona pellucida (PZP) is becoming an increasingly popular management tool and has been used in at least 76 animal species worldwide [6]. This transient contraceptive may last 10–22 months per application, depending on the formulation and species [6], [15], [16]. The management appeal of PZP is that it is expected to be reversible, is relatively easy to apply, and has few known contraindications for individuals [17], [18]. Population-level contraindications are more uncertain, but some evidence suggests decreased fecundity among untreated females, decreased fidelity in social groups, and increased adult survival can occur in populations containing PZP-treated females [19]–[22].

The individual-level efficacy and behavioral implications of PZP were previously investigated in three free-roaming feral horse populations in the western U.S. [16], [23]. We have now followed each individual female in these same populations past their targeted infertile years to further evaluate return to fertility, phenology of births, and neonate survival. We hypothesized that duration of residual infertility would be a function of cumulative historical treatments. We also hypothesized that due to the expected individual response variability to treatment and the unknown and variable duration of infertility, post-treated females would give birth later in the year compared to untreated females, and those births would be asynchronous with forage availability. We expected that parturition timing would be constrained by photoperiod and temperature and that offspring born toward the end of this temporal window would have decreased survival.

## Materials and Methods

### Ethics Statement

All data for this study were collected in accordance with the Colorado State University approved Animal Care and Use Committee protocol 03-107A-02. All data collection was conducted with the permission of the Bureau of Land Management (BLM) on public lands they administer, and involved routine observations of feral horses, which are protected under The Wild Free-Roaming Horses and Burros Act of 1971 (U.S. Public Law 91–195, as amended).

### Study Areas

The Little Book Cliffs Wild Horse Range, located in Mesa County, Colorado, U.S.A. (latitude 39°12′N, longitude 108°25′W), consisted of approximately 14,600 ha of sloping plateaus, sagebrush (*Artemisia* spp.) parks, and 4 major canyon systems. Elevations ranged from 1,500 m to 2,250 m. The study area was characterized by dense stands of Colorado piñon (*Pinus edulis*) and Utah juniper (*Juniperus osteosperma*). Population size varied from 131–179 horses during 2005–2011 and was distributed in bands of 2–9 horses. Mean annual temperature was 11.5°C (minimum = −26.7, maximum = 41.1°C). Mean total annual precipitation was 235.4 mm (range = 184.4–300.2 mm), typically falling in a monsoonal pattern of late summer rains.

McCullough Peaks Herd Management Area in Park County, Wyoming, U.S.A. (latitude 44°35′N, longitude 108°40′W), consisted of 44,400 ha of primarily open sagebrush steppe. Elevations ranged from 1,200 m to 1,964 m. Population size ranged from 169–236 horses in bands of 2–17 individuals during 2007–2011. Mean annual temperature was 8.0°C (minimum = −30.0, maximum = 37.8°C) and mean total annual precipitation was 271.2 mm (range = 168.9–389.1 mm).

The Pryor Mountain Wild Horse Range, located in Bighorn County, Wyoming and Carbon County, Montana, U.S.A. (latitude 45°04′N, longitude 108°19′W), consisted of roughly 16,000 ha of low desert, foothill slopes, forested montane slopes, steep canyons, and isolated grassy plateaus. Elevations ranged from 1,190 m to 2,625 m. Vegetation types varied greatly from lower to higher elevations of the range with lower elevations dominated by sagebrush communities, mid elevations dominated by curl-leaf mountain mahogany (*Cercocarpus ledifolius*) and Utah juniper communities, and high elevations dominated by limber pine (*Pinus flexilis*), subalpine fir (*Abies lasiocarpa*), and alpine bluegrass (*Poa alpina*). Mean annual precipitation was 161.4 mm (range = 96.7–233.4 mm) and mean annual temperature was 7.1°C (minimum = −33.9, maximum = 40.0°C). The population ranged 171–233 during the study and was arranged in bands of 2–12 individuals.

### Treatments

Free-roaming female feral horses were formerly treated with PZP for 1–5 years at all three study sites [16]. The conventional liquid form of PZP was designed to provide infertility through only a single year per inoculation, and this form was used at Pryor Mountain beginning in 2001 and at Little Book Cliffs beginning in 2002. The date of annual inoculation ranged from Jan. 5–Dec. 4, which reflects the time of year when each female was initially vaccinated, perpetuated by annual efforts to locate and re-vaccinate them remotely. These re-vaccination times varied for each horse and year due to occasional difficulties in accessing the sites as well as individual variation in horses’ tolerance of humans.

The time-release form of PZP consisted of a single inoculation of liquid PZP simultaneously applied with 3 pellets designed to release PZP in a bolus fashion at 1, 3, and 12 months, for a total of 22 months of targeted infertility [16]. All females in the treatment group at McCullough Peaks received time-release PZP in October 2004. No females were re-treated with PZP unless they had produced a foal during post-treatment. All females that were re-treated were omitted from our study at that time.

We defined post-treatment for this study as beginning two full parturition seasons after the final inoculation of conventional PZP or after the single inoculation using PZP time-release pellets. For example, if a female received a conventional PZP inoculation in autumn 2004, then she was presumably contracepted in 2005. This meant she should not produce an offspring, but could conceive at some time in 2006. The first post-treatment year would then be 2007. If that 2004 inoculation was time-release PZP, then the first post-treatment year could have been as early as 2007.

### Data Collection

We observed post-treated females from 2005 at Little Book Cliffs and Pryor Mountain and from 2007 at McCullough Peaks until they died, were removed, were treated again by managers, or the end of 2011, whichever came first. We omitted data for one female from the Little Book Cliffs and six females from McCullough Peaks because they produced offspring in every treatment year and thus were never effectively contracepted. Females that produced offspring during treatment years, but not in every year, were retained because contraception could have occurred. The untreated female group consisted of all individuals in each population that had never been inoculated with PZP and were at least 4 years old at the first year of this study period. The minimum age of post-treated females at the first year of this study period was 4 years old. The resulting data arose from observations of 88 post-treated females (age 4–24 years) and 119 untreated females (age 4–23 years) (Table 1).

**Table 1:** Parturition data from untreated and porcine zona pellucida (PZP) treated female feral horses (*Equus caballus*) at Little Book Cliffs Wild Horse Range, CO, Pryor Mountain Wild Horse Range, MT, and McCullough Peaks Herd Management Area, WY, USA.

	Little Book Cliffs WHR	Pryor Mountain WHR	McCullough Peaks HMA
	(2005–2011)	(2005–2011)	(2007–2011)
Number of untreated females	41	44	34
Number of untreated females never producing offspring	6	11	15
Age of untreated females	4–23 yr	4–15 yr	4–21 yr
Number of post-treated females	22	38	28
Number of post-treated females never producing offspring	15	18	15
Age of post-treated females	6–21 yr	4–24 yr	4–20 yr
Observed parturition range for untreated females	Feb. 23–Sep. 1	Feb. 21–Sep. 3	Jan. 15–Sep. 7
Observed parturition range for post-treated females	Mar. 5–Dec. 22	Apr. 5–Sep. 29	Feb. 20–Aug. 4
Estimated parturition peak for untreated females	May 10 (Apr. 28–May 22)	May 14 (May 4–May 24)	May 19 (May 8–May 30)
Estimated parturition peak for post-treated females	Aug. 24 (Jul. 18–Sep. 29)	Jun. 18 (May 31–Jul 6)	May 4 (Apr. 14–May 24)
Estimated difference in peak parturition date (95%CI)	105.9 (69.7–142.1) days	34.8 (17.0–52.3) days	None

Observation protocols followed Ransom et al. [16] for all three sites. For the Pryor Mountain site in 2010–2011, we also used data provided by the Pryor Mountain Mustang Center, WY, USA; these were collected under the same protocols. Throughout the study, 96.1% of all females and offspring (when present) were located at least weekly from April to October of each year. At Little Book Cliffs, 6–8 females were difficult to access weekly, but were located at least once per month. A single band containing two females at McCullough Peaks was not located weekly, but was observed at least monthly during 2007–2010. In 2011, that band was found only once and both females were observed with neonates. At Little Book Cliffs, we also used motion-activated infrared trail cameras at remote water points to provide supplemental observations. Observations during the winter were irregular and sometimes constrained by weather. It is possible that some offspring were born and died without being observed during the course of this study; however, given the intensity of observations, we believe this rarely occurred. We matched all neonates with dams based on observations of attachment (e.g., nursing, general proximity) during the early days and weeks of a neonate’s life [24]. We did not attempt to assess pregnancy in females that may have visually appeared pregnant but did not produce a viable offspring. Parturition probability was thus estimated from the frequency of live births per cohort as detected by direct observation.

We visually classified the body condition of each dam when we first detected her neonate in the field. This body condition score increased discretely from 1 to 9 as fatness increased [25]. Neonate data were collected concurrently and the general activity state of each neonate was categorized as vigorous, lethargic, or immobile. If neonates were resting when first observed, they were continually observed until the band became active again and neonate activity could be classified. Neonate date of birth was estimated subjectively by observing presence of an umbilicus, level of activity, and time elapsed since the dam was previously observed pregnant. The median time elapsed before a neonate was detected was only six days during the post-treatment observation years (*n* = 328 neonates).

### Data Analyses

#### Model structure

We used mixed-effects regression models to estimate parturition probability, phenology, and offspring survival in the maximum likelihood framework [26]. Individual female was used as a random effect on the intercept term to account for the repeated observations (multiple years) of individuals over time. This was necessary to account for variation that may be present among individuals who were sampled repeatedly, though not always equally over time. Such variation may arise from the many biotic and abiotic factors that may affect conception, pregnancy, parturition, and neonate care. We also used population as a random effect on the intercept term to account for variation attributed to location and treatment regimen. We used the lmer(⋅) function in the lme4 package in R version 2.14.1 (R Development Core Team 2011) to obtain all mixed-effects model estimates.

#### Parturition probability

Parturition history for each female was known (binomial response of the female producing at least one offspring in the past or not) from direct observation of all females <12 years old at Little Book Cliffs and Pryor Mountain, and all females <9 years old at McCullough Peaks. All older females were documented giving birth at least once within that same time period. Parturition rates of feral horses typically increase for the first few years after females reach sexual maturity, remain high through middle age, and decrease in old age [16]; therefore, we included linear and quadratic effects of age in models of parturition probability. We rescaled age in the quadratic effect by subtracting the mean age of horses (9.98 yr across all populations), so the intercept term of the model corresponded to probability of parturition at mean age.

Total annual precipitation for the biological year prior to parturition (spring of conception through spring of parturition) was used as a proxy for forage abundance, and thus potential body condition at the year of conception. Daily surface climate data were obtained from the National Climate Data Center (http://www.ncdc.noaa.gov) for Grand Junction, CO (Station 53488, about 13 km southwest of Little Book Cliffs), Cody, WY (Station 481840, about 32 km west of McCullough Peaks), and Lovell, WY (Station 485770, about 21 km south of Pryor Mountain) for all years of the study. The complete model of parturition probability included the fixed effects of age, treatment, conception year total precipitation, and parturition history. We ran a secondary linear model (lm(⋅) function) for only the post-treated females that produced offspring in order to investigate the influence of age at first treatment and number of treatments received (1–5 annual inoculations). This secondary model only applied to horses at Little Book Cliffs and Pryor Mountain, where repeated inoculations of conventional liquid PZP were applied.

#### Parturition phenology

We hypothesized that photoperiod and temperature were critical factors influencing phenology of conception, and thus phenology of parturition. Consequently, the model of parturition phenology included treatment and temperature at the approximate conception date as fixed effects, and individual and population as random effects on the model intercept. A supplemental model was considered for post-treatment females at Little Book Cliffs and Pryor Mountain to assess the interaction of last treatment date and number of consecutive annual treatments as a fixed effect, also using individual and population as random effects. Because only one treatment was applied at McCullough Peaks and the actual decay rate of the time-release pellets was unknown, we could not apply this supplemental model for that site.

#### Survival

Survival was a binomial response attributed to persistence of a neonate from parturition until the following year April observation or death during that time period. Managers removed 30 neonates from the range during their birth year, and those animals were not included in modeling survival. We hypothesized that survival was a function of treatment, dam age, dam body condition, mean winter temperature (Nov. 1–Mar. 31), and the temporal difference between birth date and spring peak available forage. We used Normalized Difference Vegetation Index (NDVI) data obtained from the National Aeronautics and Space Administration (http://modis.gsfc.nasa.gov) and reconciled using ArcGIS software (Esri, Redlands, California) to assess temporal variation in forage availability. The phenology product from Tan et al. [27] was used to generate date of mean maximum NDVI in each year across each study area. Mean NDVI was used as a temporal indicator of forage availability, but not forage abundance because tree presence and distribution can strongly influence magnitude of this metric.

## Results

### Parturition Probability

During the years of observation, 15–44% of untreated females and 47–68% of post-treated females never produced offspring (Table 1). Of all post-treated females in the study, 81% had successfully produced offspring prior to treatment and the remaining 19% were <4 years old when first inoculated. Likewise, 97% of all untreated females in the study had successfully produced offspring prior to or during the study period. The probability of producing offspring was strongly related to treatment (*z* = −6.90, *P*<0.001) and age (*z* = −2.93, *P* = 0.003), and weakly influenced by total annual precipitation during year of conception (*z* = 1.79, *P* = 0.074). At mean age and mean conception-year precipitation, the estimated parturition probability for post-treated females was 25.6% (95% CI = 17.8–35.2%) and for untreated females was 64.1% (53.1–73.9%) if they had produced offspring in any year prior to the study. The estimated parturition probability was only 2.1% (1.3–3.2%) for post-treated females and 10.7% (7.0–15.9%) for untreated females that had never previously produced offspring. Parturition probability increased 0.497% (0.494–0.500%) per year of age for all females until age 10; after which it declined at the same rate. Population contributed to a small amount of variation in the model (σ = 0.33) but individual identity was more influential (σ = 0.82).

**Figure 2:**
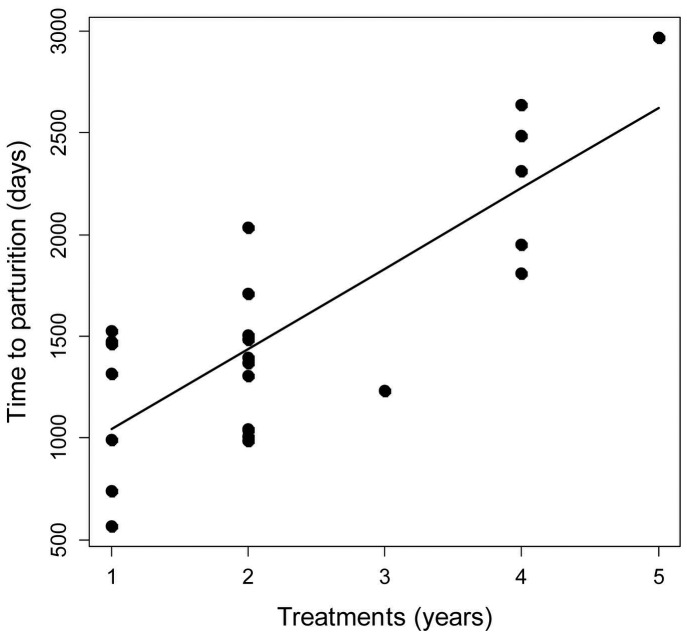
Time between final contraceptive inoculation and first parturition from feral horse (*Equus caballus*) females. Births shown are from horses at Little Books Cliffs Wild Horse Range, CO, USA and Pryor Mountain Wild Horse Range, MT, USA as a function of consecutive annual porcine zona pellucida (PZP) inoculations, 2005–2011.

Of the 52 females that received 1–5 annual injections of conventional PZP (Little Book Cliffs and Pryor Mountain), 27 produced at least one offspring post-treatment. The length of time between last inoculation and first parturition ranged from 565–2,971 days and was strongly influenced by the total number of years a female was treated (*t* = 5.18, *P*<0.001). Estimated time to parturition increased 411.3 (246.5–576.0) days per year of consecutive treatment (Figure 2). Neither age at first treatment (*t* = −1.04, *P = *0.309) nor fertility history (*t* = 0.01, *P = *0.991) influenced the length of time to parturition. The secondary model was not run for McCullough Peaks, where all 36 females were treated with the 22-month time-release PZP pellets on the same day. Thirteen post-treated females in that population produced an offspring during the 5 years of observation and the observed length of time between inoculation and first parturition ranged from 530–2,000 days.

### Parturition Phenology

**Figure 3:**
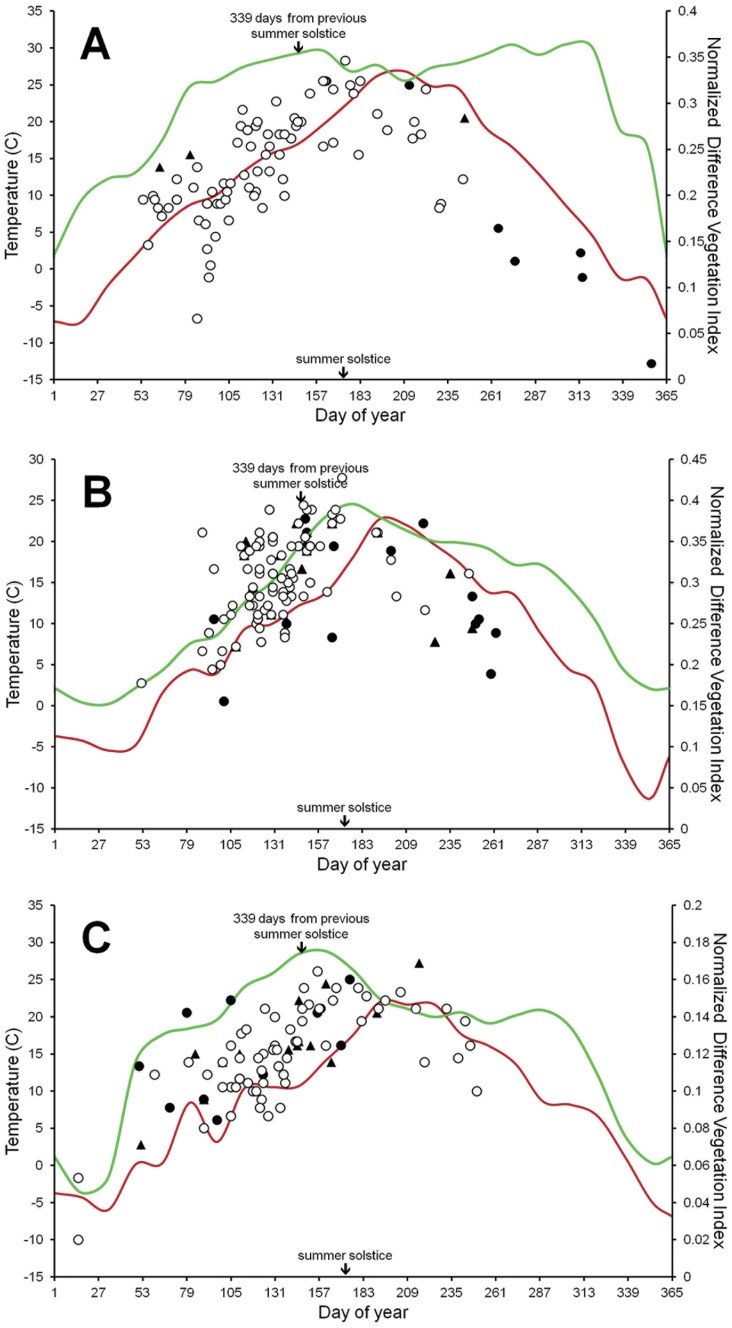
Birth phenology of feral horse (*Equus caballus*) untreated (○) and post-treated (• = first post-treatment birth, ▴ = birth subsequent to •) females. Births are shown as a function of temperature at approximate conception date for populations at Little Book Cliffs Wild Horse Range, CO (A), 2005–2011, and Pryor Mountain Wild Horse Range, MT (B), 2005–2011, and McCullough Peaks Herd Management Area, WY (C), 2007–2011. Post-treated females were previously inoculated with the immunocontraceptive porcine zona pellucida (PZP). Mean Normalized Difference Vegetation Index (green) represents temporal availability of forage. Mean daily surface temperature is shown in red.

Births from all untreated females increased in frequency toward the summer solstice and decreased toward the winter solstice, and those trends corresponded with temperature and forage availability (Figure 3). Parturition from untreated females was similar between populations and ranged from Jan. 15–Sep. 7 (Table 1). Parturition from post-treated females ranged from Feb. 20–Dec. 22, and the estimated peak was 31.5 (17.0–46.0) days later than births from untreated females, after controlling for temperature at conception date. The latest neonate born to a post-treated female arrived 7.5 months after the peak in births from untreated females, indicating conception occurred within 24–31 days of the winter solstice. Among the post-treated females that gave birth late and then did not produce a neonate the next year, there was some evidence that subsequent births began to shift back toward the phenology observed for untreated females (Figure 3).

### Survival

Nearly every neonate born was observed in a vigorous activity state, with the exception of four that were immobile (one to a post-treated female) and two that were lethargic (none to post-treated females). All six of those neonates died before the following spring. Body condition of dams at first observation of neonates ranged 3–9 (mean = 5.73, 5.61–5.84). Eighty-five percent of neonates were observed alive in the spring following their birth. The estimated survival probability of a neonate born to a dam in mean body condition at spring peak greenness was 79.9% (67.9–85.4%). This probability decreased 11.4% (10.7–12.0%) with each 1-unit decrease in dam body condition. Survival declined 1.4% (1.4–1.5%) for every 10 days after peak greenness that parturition occurred.

Treatment (*z* = −1.49, *P* = 0.137), band size (*z* = 0.67, *P* = 0.500), dam age (*z* = −1.16, *P* = 0.247), and mean winter temperature (*z* = 0.89, *P* = 0.372) did not influence survival in this model; however, the treatment and winter temperature effects were disparate between sites. At Little Book Cliffs and Pryor Mountain, treatment weakly influenced survival (*z* = −1.78, *P* = 0.075), whereas at McCullough Peaks it clearly had no influence (*z* = 0.01, *P* = 0.993). Likewise, mean winter temperature influenced survival (*z* = −2.16, *P* = 0.031) at Little Book Cliffs and Pryor Mountain, but not at McCullough Peaks (*z* = 1.00, *P* = 0.320).

## Discussion

### Parturition Probability

The probability of post-treated females producing offspring was 38.5% lower than for untreated females, after controlling for differences due to age, precipitation during conception year, and fertility history. The return to fertility rates observed were highly variable and this may in part be due to the disparate abilities of individual females to raise therapeutic-level antibodies against PZP and the length of time those antibodies persisted above threshold concentrations [15], [28], [29]. A similar result was reported for horses at Assateague Island National Seashore, MD and VA, USA, where 68.8% of 32 female horses treated for three consecutive years with PZP became pregnant 1–4 years after the last treatment [8]. Only 3 horses were vaccinated for four consecutive years in that study: one became pregnant 3 years after the last treatment, another became pregnant after 4 years, and the third became pregnant after 8 years. In a similar, but critically endangered species, *E. ferus przewalskii*, only 45% of 20 females that had been inoculated with PZP for 3 consecutive years demonstrated fertility after 6.7 post-treatment years [30]. More surprisingly, 4 of 9 females in that study that had only received a single treatment (one primer injection followed by one booster injection) never produced any offspring after 4–8 years of observation. While the mechanism for explaining this protracted infertility in *E. ferus przewalskii* is unknown, some of the Assateague horses were found to have experienced ovulatory failure [31]. This is not the targeted physiological response of PZP vaccination, and the effect was highly variable between individuals due to the episodic nature of ovulatory failure, as opposed to a chronic condition. Studies of non-equid species have demonstrated atrophic changes in ovarian morphology, folliculogenesis, and reproductive endocrine function, indicating that prevention of sperm binding may not be the only mechanism acting on fertility in PZP-inoculated animals [32]–[35].

### Parturition Phenology

Parturition phenology for North American feral horses has been shown to peak during May [36]–[38]. We estimated parturition from untreated females peaked May 10–19 across the 3 populations, which was 22–43 days before spring forage availability began to decline, and placed conception roughly 7–15 days before the longest day of sunlight (or 323–332 days from previous summer solstice). This phenology appears synchronized with the most abundant forage during the parturition period while females’ metabolic needs should be elevated from late term pregnancy and lactation [39]. Our findings for PZP-inoculated females demonstrated a markedly different phenology. The estimated peak in parturition from post-treated females at two sites (Aug. 24 at Little Book Cliffs and Jun. 18 at Pryor Mountain) occurred as forage availability was declining; however at Little Book Cliffs where births were latest, the monsoonal rains provided a bimodal distribution in greenness (Figure 3A). The second peak in forage availability arrived Nov. 1, which was 69 days after estimated peak in parturition from post-treated females.

This protracted birth phenology resulted in parturition dates spanning 305 days for post-treated females as compared to 229 days for untreated females. Across all females in the 3 populations, the parturition season ranged nearly the entire year (341 days). This indicates surprising plasticity in birth phenology of temperate latitude horses given the physiological mechanisms thought to be driving reproductive cyclicity. At Shackleford Banks, North Carolina, USA, female feral horses exhibited estrus beyond the normal breeding season after being vaccinated annually with PZP for 1–6 years [38]. Based on the observed birth phenology in our study, this phenomenon occurred in our subjects as well; however, we recorded 81.4% of 328 documented births between March 1 and June 21. This suggests that photoperiod and temperature remain fundamentally important in regulating birth phenology, but several other mechanisms may be acting to help explain the wide range of birth dates observed. Follicular activity of the estrous cycle during the first half of the breeding season is characterized by more numerous large follicles and greater incidence of anovulatory waves [40]. Gestation length is variable and has been attributed to seasonal variations that influence nutrition of females [41]. The annual rhythm of luteinizing hormone (LH) secretion is partially regulated by photoperiod, but also has a strong endogenous component [42]. Age and fertility history of females can strongly influence the occurrence of a winter anovulatory period [43], as can melatonin sensitivity of individuals [2]. This litany of influences is compounded by the known variations in PZP immunocontraception efficacy and duration, as well as the uncertain mechanism of action that may be confounded with ovarian pathologies.

The protracted breeding season of PZP-vaccinated females found in our study, as well as by Nuñez et al. [38], indicates that horses that would normally conceive during their first or second reproductive cycle of the year continue to cycle throughout the year and then only conceive when contraception sufficiently decays. This inherently extends the breeding season and challenges the physiological constraints assumed to be regulating fertility. The ‘self-correcting’ trend we observed provides additional empirical support for the importance of such abiotic factors in birth phenology, but also illustrates that phenological shifts associated with the uncertainties surrounding immunocontraception may be short term. The long term effects of PZP treatment on population ecology are less clear given the behavioral components that can affect social networks, as well as fitness of untreated females in social groups [19], [21]–[23].

It should be noted that the protracted phenology detected at Little Book Cliffs and Pryor Mountain was not observed at McCullough Peaks, where the final bolus release of PZP is assumed to have occurred around July. This should have led to infertility persistence through the following July but appears to have persisted much longer in many individuals. Some variation in parturition phenology likely arose from the unique immune response of each female to the vaccine, but this was not compounded by the widely disparate vaccination dates experienced at the other two sites, and thus a more consistent phenology was observed.

### Survival

The ultimate consequence of altered birth phenology is survival. Collectively, survival of all neonates in our study was consistent with the mean survival rate (73.9%) derived from 12 historic studies on feral horse neonates [44]. Survival probability for neonates in our study was 79.9%, at mean body condition of the dam and at spring peak NDVI. This probability predictably decreased with decreasing dam body condition and with temporal distance between date of birth and spring peak NDVI. Treatment did not influence survival in the global model, but was influential at the two sites where unseasonably late births were observed. The treatment effect on survival at Little Book Cliffs was somewhat ameliorated by the secondary peak in forage availability following the monsoons. Such fine scale ecosystem effects may be important considerations when assessing the potential for fertility control applications on specific populations.

### Conclusion

Humans are increasingly attempting to manage the planet’s wildlife and habitats with new tools that are often not fully understood. The transient nature of the immunocontraceptive PZP can manifest into extraordinary persistence of infertility with repeated vaccinations, and ultimately can alter birth phenology in horses. This persistence may be of benefit for managing overabundant wildlife, but also suggests caution for use in small refugia or breeding facilities maintained for repatriation of rare species. These results introduce ethical questions toward regulating populations with tools that may alter ecology of the species, but also offer quantitative insights that can be weighed with the depth of management need. The growing need for humane, non-lethal, population control measures also led us to empirical evidence that illustrates the physiological flexibility of horse reproduction in the race for fitness. Photoperiod and temperature are powerful inputs driving the biological rhythms of conception and birth in horses, but do not limit the ability of horses to conceive under perturbed conditions.
